# Transmission of New Bovine Prion to Mice

**DOI:** 10.3201/eid1207.060107

**Published:** 2006-07

**Authors:** Thierry G.M. Baron, Anne-Gaëlle Biacabe, Anna Bencsik, Jan P.M. Langeveld

**Affiliations:** * Agence Française de Sécurité Sanitaire des Aliments, Lyon, France;; † Central Institute for Animal Disease Control, Lelystad, the Netherlands

**Keywords:** Bovine spongiform encephalopathy, prion, cattle, dispatch

## Abstract

We previously reported that cattle were affected by a prion disorder that differed from bovine spongiform encephalopathy (BSE) by showing distinct molecular features of disease-associated protease-resistant prion protein (PrP^res^). We show that intracerebral injection of such isolates into C57BL/6 mice produces a disease with preservation of PrP^res^ molecular features distinct from BSE.

Until recently, transmissible spongiform encephalopathy (TSE) in cattle was believed to be caused by a single strain of infectious agent identified at the beginning of a foodborne epidemic of bovine spongiform encephalopathy (BSE). Characterization of the infectious agent associated with BSE showed unique features. These include defined incubation periods and distribution of brain lesions after transmission to wild-type mice, not only directly from cattle, but also after natural or experimentally induced cross-species transmission (*1**,**2*). The uniform features of the disease in cattle have also been shown by analysis of the distribution of neurodegenerative brain lesions at different places during the BSE epidemic (*3**,**4*).

Western blot analyses of protease-resistant prion protein (PrP^res^) accumulating in the brains of animals and humans with BSE have demonstrated specific molecular features. These include a low molecular mass of unglycosylated PrP^res^ with high proportions of diglycosylated PrP^res^ (*5**,**6*). However, recent studies reported cases of prion abnormalities in cattle with different PrP^res^ features (*7**,**8*). Three cattle isolates from France have been reported, characterized by a higher apparent molecular mass of unglycosylated PrP^res^ (H-type isolates) and decreased levels of diglycosylated PrP^res^ when compared with BSE isolates (*7*). In addition, only PrP^res^ from H-type isolates were labeled by monoclonal antibody P4 with defined PrP^res^ N terminus epitope specificity, in contrast with PrP^res^ from BSE isolates, which suggests a different cleavage by proteinase K of the disease-associated protein (*9*).

Twenty years after identification of the BSE epidemic in cattle, the origin of the BSE agent remains controversial (*10**,**11*). Researchers have often considered the most likely source to be a recycled infectious agent derived from prion-associated diseases found in other species, such as scrapie in sheep and goats. The recent description of unusual phenotypes of bovine prion diseases distinct from BSE is therefore puzzling (*7*). This situation has been reinforced by a second bovine amyloidotic spongiform encephalopathy found in cattle in Italy (*8*). However, whether such cases of bovine prion disorders were transmissible, and to what extent the infectious agent caused specific features distinct from BSE, have not been demonstrated.

## The Study

Experimental groups of 20 (4- to 6-week old) C57BL/6 female mice (Charles River, L'Arbresle, France) were injected intracerebrally with 20 μL of 10% (weight/volume) homogenates per mouse prepared from brain stem samples of 3 cattle TSE isolates. Two of the isolates were characterized, as previously described (*7*), by a higher molecular mass of unglycosylated PrP^res^ (H-type isolates) and labeling with P4 monoclonal antibody (Table). A typical cattle BSE isolate was also analyzed. Mice were housed and cared for in an appropriate biohazard prevention area (A3) according to European (directive 86/609/EEC) and French ethical committee (decree 87–848) guidelines. Mice were checked at least weekly for neurologic clinical signs and were killed when they exhibited signs of distress or confirmed evolution of clinical signs. The whole brain of every second mouse was frozen and stored at –80°C before Western blot analysis. The other brains were fixed in 4% paraformaldehyde for other histopathologic studies.

**Table:** Cattle sources of transmissible spongiform encephalopathy (TSE) used for experimental infections of C57BL/6 mice and transmission results*

Cattle TSE isolate	Age, y	Breed	Molecular type	Survival periods (d) in C57BL/6 mice (mean ± SD)	Western blot results†
1	8	Charolais	H	702 ± 117	8/9
2	12	Crossbreed	H	652 ± 85	10/10
3	4	Prim'Holstein	Typical	511 ± 89	8/9

*SD, standard deviation. †No. mice positive for disease-associated prion protein/no. mice analyzed.

Frozen mouse brain tissues and fixed brain tissues were examined by Western blot analysis and immunohistochemical tests as previously described (*12**,**13*). PrP^res^ extracted from half of whole brain was detected with monoclonal antibodies Sha31 (1:10 from TeSeE sheep/goat Western blot, Bio-Rad, Hercules, CA, USA) (*14*) and 12B2 (340 ng/mL) (*15*). These antibodies are directed against the 144-WEDRYYRE-151 and 88-WGQGG-92 murine amino acid PrP sequences, respectively. Antibody 12B2, which has an N-terminal specificity similar to that of monoclonal antibody P4, shows poor binding to BSE-derived PrP^res^, but unlike P4, binds with high affinity to prion protein from most mammalian species, including mice and cattle. Bound antibodies were detected by using enhanced enzymatic chemiluminescence (Amersham, Little Chalfont, UK) or Supersignal (Pierce, Rockford, IL, USA) and visualized either on film (Biomax, Eastman Kodak, Rochester, NY, USA) or directly in an image analysis system (Versadoc, Bio-Rad). Molecular masses of PrP^res^ glycoforms were determined as the average of the center positions of the bands from at least 3 repeated electrophoretic procedures, as measured by comparison with a biotinylated marker (B2787, Sigma, Saint Louis, MO, USA) included on each gel. Immunologic reactivities of antibodies 12B2 and Sha31 were compared in Western blots run in parallel with the same samples with both antibodies.

After intracerebral injection of cattle brain samples into C57BL/6 mice, disease was observed in mice with the 2 H-type isolates, as well as with the BSE sample. Survival periods of mice and results of PrP^res^ detection among mice analyzed by Western blot are shown in the Table.

**Figure 1:**
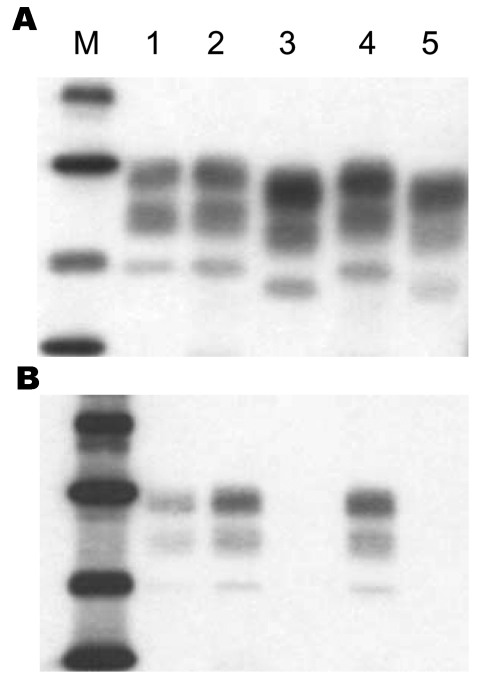
Western blot analysis of disease-associated prion protein (PrP^res^) from proteinase K–treated brain homogenates of C57BL/6 mice infected with type H (lanes 2 and 4) or bovine spongiform encephalopathy isolates (lanes 3 and 5). PrP^res^ of mice infected with an experimental scrapie strain (C506M3) (*6*) was used as a control (lane 1). Monoclonal antibodies used for detection of PrP^res^ were Sha31 in panel A and 12B2 in panel B. Lane M, molecular mass markers: 39.8, 29, 20.1, and 14.3 kDa.

Western blot analysis of PrP^res^ from H-type–infected mouse brains in comparison with BSE-infected mice is shown in Figure 1. All positive mice in the same experimental group showed the same Western blot pattern. This pattern showed higher molecular mass PrP^res^ glycoforms in mice infected with H-type isolates than in mice infected with a typical BSE agent (1.1- to 1.5-Da difference in the unglycosylated PrP^res^ (Figure 1A). Studies of PrP^res^ protease cleavage showed that only the PrP^res^ of mice infected with H-type isolates was recognized by antibody 12B2 (Figure 1B). This finding is in contrast to the result obtained with monoclonal antibody Sha31 directed against an epitope in the central region of the protein, which showed that the 12B2 epitope was preserved in H-type–infected mice. Thus, the molecular features of H-type cattle isolates, which are distinct from those of the BSE agent, were maintained after development of disease in mice.

**Figure 2:**
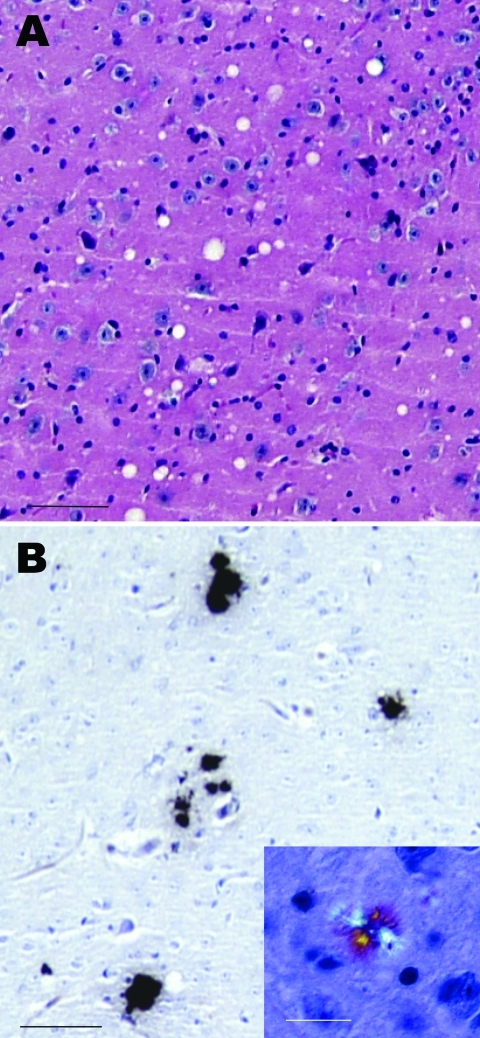
Histopathologic analysis of brain of a C57BL/6 mouse infected with a type H isolate. A) Characteristic vacuolar lesions in the thalamus (hematoxylin and eosin stained, scale bar = 60 μm). B) Immunohistochemical analysis of prion protein with monoclonal antibody 12B2 (diluted 1:200) shows the absence of granular deposition, but the presence of plaques in the thalamus. The inset shows that plaques are amyloids since they bind Congo red and show birefringence in polarized light (scale bar = 60 μm, scale bar in inset = 16 μm).

Histopathologic analysis showed vacuolar lesions in the thalamus (Figure 2A) that were absent from the hypothalamus, cochlear nucleus, and superior collicules. These 3 neuroanatomic sites were severely affected in C57BL/6 mice brain after primary passage of the BSE agent as we and others have reported (*1*). Abnormal PrP was detected only in amyloid plaques (Figure 2B), in contrast to what was reported after BSE transmission in C57BL/6 mice (*1*).

## Conclusions

Our data show that the recently identified bovine H-type isolates involve an infectious agent that can induce development of a disease across a species barrier, while maintaining the specific associated PrP^res^ molecular signature. This evidence in favor of a new bovine prion strain in cattle suggests that BSE is not the only transmissible prion disease in cattle. The origin of such cases has not been determined (*7*). These cases suggest either the existence of alternative origins of such diseases in cattle or phenotypic changes of PrP^res^ after infection with the BSE agent. However, based on analysis of molecular features of prion diseases in cattle, this situation is similar to that in humans (*5*), in which different subtypes of sporadic Creutzfeldt-Jakob disease agents are found.
